# Heterogeneous effects of physical activity on physiological stress during pregnancy

**DOI:** 10.1371/journal.pdig.0000837

**Published:** 2025-10-22

**Authors:** Jenifer Rim, Qi Xu, Xiwei Tang, Tamara Jimah, Yuqing Guo, Annie Qu

**Affiliations:** 1 Department of Statistics, University of California, Irvine, California, United States of America; 2 Department of Statistics and Data Science, Carnegie Mellon University, Pittsburgh, Pennsylvania, United States of America; 3 Department of Mathematical Sciences, University of Texas at Dallas, Richardson, Texas, United States of America; 4 Department of Public Health and Health Sciences & Department of Pharmacy and Health Systems Sciences, Northeastern University, Boston, Massachusetts, United States of America; 5 Sue & Bill Gross School of Nursing, University of California, Irvine, California, United States of America; 6 Department of Statistics and Applied Probability, University of California, Santa Barbara, California, United States of America; Iran University of Medical Sciences, IRAN, ISLAMIC REPUBLIC OF

## Abstract

Pregnancy involves rapid physiological and psychological changes that can increase vulnerability to health complications, underscoring the need for timely, individualized support. Mobile health (mHealth) tools offer a scalable way to capture repeated measures of health status throughout pregnancy, facilitating longitudinal assessment and the opportunity for timely intervention. This study leveraged mHealth technologies, including the Oura smart ring and ecological momentary assessment (EMA) via a mobile app, to examine how emotional distress affects the relationship between physical activity (PA) and heart rate variability (HRV), an indicator of physiological stress during pregnancy. Specifically, we examined whether emotional distress, measured via daily EMA surveys, moderates the association between physical activity and nighttime HRV, captured by continuous Oura ring data. Hence, this analysis integrated temporally aligned wearable and self-report data to investigate the interaction between subjective emotional states and objectively measured physical activity patterns. Consenting participants, aged 18–40 years, with a healthy singleton pregnancy in the second trimester, were enrolled in the study. Our findings revealed that on days with high emotional distress, each additional 1,000 steps was associated with a 3.5% increase in nighttime HRV (*p*-value < 0.001; 95% CI: 2.6%, 4.4%). In contrast, physical activity had little to no association with HRV on days with moderate distress (0.6%; 95% CI: -0.7%, 1.9%) and low distress (0.6%; 95% CI: -0.4%, 1.5%). These findings suggest that physical activity may be particularly beneficial on high-distress days, supporting the development of adaptive interventions that prioritize PA engagement during periods of elevated emotional distress. Based on our model-estimated moderation effects, we may recommend that a pregnant woman increase her physical activity on high-distress days due to a strong positive PA-HRV association, whereas for those who do not experience much emotional distress, the recommendation may be less emphasized, given the weaker observed association.

## Author summary

Pregnancy involves significant physiological and psychological changes that impact maternal and fetal health. Heart rate variability (HRV), a key biomarker of autonomic function and cardiovascular health, reflects the body’s ability to regulate physiological stress. In this study, we combined wearable sensor data from the Oura smart ring with daily self-reported measures of emotional distress to examine how physical activity relates to nighttime HRV. We observed that physical activity was most strongly associated with higher HRV on days when participants reported greater emotional distress, whereas little to no association was found on low and moderate-distress days. These findings highlight the potential value of promoting physical activity during periods of heightened emotional distress as a strategy to support physiological stress regulation during pregnancy.

## Introduction

Pregnancy involves dynamic physiological and psychological changes that can increase vulnerability to stress-related health risks for both the mother and fetus. Physiological stress, resulting from changes in autonomic and endocrine function, has been linked to adverse outcomes such as preterm birth, low birth weight, and developmental issues in the child [1,2], as well as an increased risk of maternal conditions such as hypertension, gestational diabetes, and postpartum depression [3]. Heart rate variability (HRV), a noninvasive measure of autonomic nervous system function, provides an objective indicator of physiological stress and the body’s capacity for recovery [4,5]. With the growing availability of mobile health (mHealth) technologies, HRV can now be monitored continuously in naturalistic settings, enabling the study of how daily behavioral and emotional factors influence maternal stress regulation. Understanding these modifiable factors is essential for developing timely, personalized interventions to support maternal and fetal health.

HRV refers to the variation in time intervals between heartbeats and reflects the balance between sympathetic (stress-related) and parasympathetic (relaxation-related) nervous system activity [6]. Higher HRV serves as a critical indicator of autonomic nervous system function, generally associated with good cardiovascular health and resilience to stress. Alternatively, reduced HRV indicates compromised autonomic regulation and may suggest reduced capacity for physiological stress regulation [5]. Thus, HRV not only reflects physiological stress resilience but also serves as an indicator of cardiovascular fitness, both of which are important for understanding and informing pregnancy health. This is particularly relevant during the second trimester, as this period is marked by significant cardiovascular and physiological adaptations [7,8].

Various factors contribute to fluctuations in HRV. Notably, physical activity (PA) has been widely studied for its beneficial role in enhancing HRV. Prior studies reveal that regular PA improves autonomic function by boosting HRV and facilitating physiological stress adaptation [9,10], which in turn can enhance the body’s ability to manage stress [11]. Particularly, PA during pregnancy has been associated with maternal and fetal health benefits, including enhanced psychological well-being [12].

It is important to distinguish between emotional distress and physiological stress, as these related but distinct constructs influence HRV differently. Emotional distress refers to self-reported negative emotional states, such as sadness or anger, whereas physiological stress reflects autonomic nervous system changes, which can be measured objectively using HRV. Emotional distress has been linked to reduced HRV due to its impact on autonomic regulation, indicating a diminished capacity for physiological stress adaptation [13,14]. Moreover, emotional distress can trigger physiological responses that further alter autonomic function and influence maternal and fetal health [15].

Synthesizing these findings highlights how PA and emotional distress jointly relate to HRV. The relationship between PA and HRV is hypothesized to be moderated by emotional distress, with the beneficial effects of PA amplified on days of higher emotional distress, consistent with the buffering effect framework, which proposes that behaviors like PA can protect against the harmful effects of distress [16]. A key mechanism is vagal tone, a marker of parasympathetic activity reflecting the vagus nerve’s role in regulating heart rate and promoting recovery [17]. Emotional distress is associated with reduced vagal tone [18], whereas PA can enhance vagal activity [19], potentially counteracting distress-related reductions in HRV and restoring autonomic balance.

Advancements in mobile health (mHealth) technologies now enable continuous monitoring of HRV and other physiological markers while also capturing psychological assessments throughout pregnancy [20]. Wearable devices, such as the Oura smart ring, provide objective data on heart rate, HRV, PA, and sleep, allowing longitudinal tracking of maternal health trends outside traditional clinical and laboratory settings [21]. In conjunction, ecological momentary assessment (EMA) collects real-time self-reports of emotional distress, capturing the psychological experiences of participants in their natural environment [22]. Integrating these approaches offers a more comprehensive assessment of both objective (physiological measures) and subjective (psychological states) factors that influence HRV during pregnancy.

Despite prior studies linking physical activity and emotional distress separately to HRV, most rely on static, cross-sectional measures, limiting the ability to capture how day-to-day variation in emotional distress may change the physiological benefits of PA [23]. In pregnancy, where both distress and behavior fluctuate continuously, this lack of temporal resolution constrains understanding of within-person dynamics. Consequently, the moderating role of emotional distress on the PA-HRV relationship remains underexplored, highlighting a critical gap that continuous, real-world mHealth data can address.

To address this knowledge gap, this study aims to conduct a comprehensive analysis integrating both objective and subjective measures to examine their influence on HRV during pregnancy. Specifically, we aim to (1) examine how emotional distress and PA individually affect HRV, while adjusting for other factors such as age, pre-pregnancy body mass index (BMI), heart rate, and sleep parameters (deep and REM sleep), which are also known to influence HRV [24–27], and (2) examine whether daily emotional distress moderates the association between PA and HRV during pregnancy. Based on buffering effect theory [16], we hypothesize that the positive association between PA and HRV will be stronger on days when emotional distress is elevated.

By integrating objective physiological data from wearable devices with subjective self-reports of emotional states, this study aims to enhance the understanding of the complex interactions between PA, emotional distress, and HRV in pregnant women. Ultimately, our findings could inform intervention strategies, encouraging PA as a potential means to regulate HRV during pregnancy and promote overall maternal and fetal health.

## Materials and methods

### Ethics statement

This study was reviewed and approved by the University of California, Irvine Institutional Review Board (UCI IRB; Approval Number: 20195363). All procedures were conducted in accordance with the approved protocol and relevant regulations. Written informed consent was obtained from all participants prior to their participation in the study.

### Study design and sampling

The study used a longitudinal observational design. We chose a longitudinal design to capture within-person fluctuations in physical activity, emotional distress, and HRV across multiple days. This longitudinal design is also motivated by the nature of mobile health data, which allows us to collect abundant and dynamic information from each individual. Such data offer high-resolution, temporally rich, and personalized records. At the same time, it is relatively common in this type of study to have a limited number of participants. Therefore, it is important to leverage the longitudinal data collected from the same individuals to maximize the analytical power and extract meaningful insights. Moreover, this approach allowed us to examine temporal variation and dynamic interactions between variables, rather than relying on static or between-subject comparisons.

Following IRB (Institutional Review Board) approval, convenience sampling was employed to recruit participants by disseminating the study flyer through established local community partners working with underserved perinatal women in the Southern California region. We acknowledge that this approach may limit generalizability due to self-selection bias. The inclusion criteria were pregnant women aged 18–40 years, with a healthy singleton pregnancy, and access to a smartphone. The research coordinator explained the study procedure and obtained consent from participants using the IRB-approved Study Information Sheet. Once consent was obtained, the Oura ring wearable device was shipped to participants for data collection.

A total of 20 participants were eligible for the Oura ring data collection, but two withdrew before the data analysis stage due to family circumstances, leaving 18 who provided data on physiological metrics such as HRV and resting heart rate. More detailed information on these participants can be found in an earlier study [28]. Meanwhile, 17 participants provided EMA data through smartphone surveys, capturing self-reported emotional experiences in real time. Among them, only 13 had both Oura ring and EMA data. We retained 9 participants by selecting those with second trimester data and aligning the merged dataset, pairing each day’s Oura ring data with the previous day’s EMA data, to examine how daytime emotional states influenced subsequent nighttime physiological metrics.

Our focus on the second trimester was guided by both methodological and biological considerations. The second trimester typically provides a more stable baseline for observing physiological and behavioral patterns compared to the first and third trimesters, where variation in HRV or distress levels is affected by many other factors, making it difficult to measure the effect of physical activity accurately (e.g., nausea in early pregnancy or sleep disturbances in late pregnancy). Restricting analyses to the second trimester reduced extra heterogeneity in gestational timing and minimized confounding due to trimester-specific physiological changes. This design choice allows us to isolate the relationship between physical activity, emotional distress, and HRV during a relatively stable developmental window.

### Data collection

Self-reported demographic data, including maternal age, ethnicity, and pre-pregnancy body mass index (BMI), were collected using REDCap, a secure data collection platform [29]. The Oura ring, a waterproof wearable device equipped with multiple sensors, measured physiological signals through an optical pulse waveform detected from the participant’s finger, including heart rate variability (HRV), heart rate, and physical activity [30]. The ring automatically transferred data to an app on the participant’s smartphone via Bluetooth, delivering a daily summary. Validation studies have shown the Oura ring’s accuracy in comparison with gold standard tools such as polysomnography, electrocardiography, and medical-grade actigraphy [31–34]. These studies support the Oura ring’s validity for measuring nighttime HRV, resting heart rate, and daily step counts in naturalistic settings.

Data on the participants’ various emotional states during pregnancy were also collected using daily ecological momentary assessment (EMA), a method used to capture real-time emotional states or moods by prompting participants to report their feelings during the day [22]. EMAs were delivered to participants daily over the course of the study at approximately noon via a mobile application. Responses were automatically saved and subsequently retrieved from the study dashboard for analysis. A description of the methods employed in developing the app-based platform can be found in an earlier study [21]. Specifically, the EMA prompts solicited information regarding participants’ current emotional states, encompassing both positive (healthy, cheerful, content, excited, safe) and negative (sad, lonely, angry, overwhelmed, stressed, tired, worried) feelings. By collecting data in real-time, EMA minimizes recall bias and captures fluctuations in emotional states as they occur, allowing for a better understanding of how emotions vary for an individual [35]. EMA response rates ranged from 10% to 100% across participants. To assess the robustness of our findings, we conducted a sensitivity analysis excluding participants with response rates below 40%. The results remained consistent, with similar effect sizes and direction of associations. Physiological measures, such as HRV and resting heart rate, were collected overnight and reported at the start of each day, while emotional states were recorded later in the day via EMA.

### Data preprocessing

In this study, we focused on the second trimester (clinically, 14–28 weeks gestation), a period characterized by significant physiological and cardiovascular changes [7,8] but relatively greater emotional stability compared to the third trimester.

For our analysis, we combined the EMA data with physiological data from the Oura ring to jointly examine the relationship between emotional and physiological factors, as well as their interactions, on HRV during pregnancy. To capture the potential carryover effect of daytime emotional distress on physiological stress regulation during sleep, we paired each day’s EMA-reported emotional distress (completed during the day before bedtime) with the HRV recorded during that night. This temporal ordering is consistent with prior research suggesting that psychological states earlier in the day can influence overnight autonomic recovery [36]. Aligning data in this way allows us to examine whether daytime emotional distress moderates the association between daytime physical activity and subsequent nighttime HRV. After data preprocessing, we retained 9 subjects, with an average of 10 daily observations per subject (93 daily observations total). Although the final sample size is limited, this study was designed as a preliminary individualized investigation to assess day-level interactions among physical activity, emotional distress, and HRV during pregnancy. While the number of subjects is limited, the longitudinal design allows for the detection of consistent within-person trends.

In the daily EMA survey, a total of 12 emotions were evaluated, ranging from positive to negative emotions. Positive emotions are characterized by feelings of well-being and happiness, such as being cheerful, excited, healthy, content, and safe, while negative emotions involve states of distress and discomfort, including feeling tired, overwhelmed, stressed, worried, angry, lonely, or sad. The intensity of each emotion was assessed using a 5-point Likert scale [37] with values ranging from 0 (Not at all) to 4 (A lot). For example, participants were asked:

“How sad are you feeling right now?”

Response options:

0 (Not at all),

1 (A little),

2 (Somewhat),

3 (Quite a bit), or

4 (A lot).

**Fig 1:**
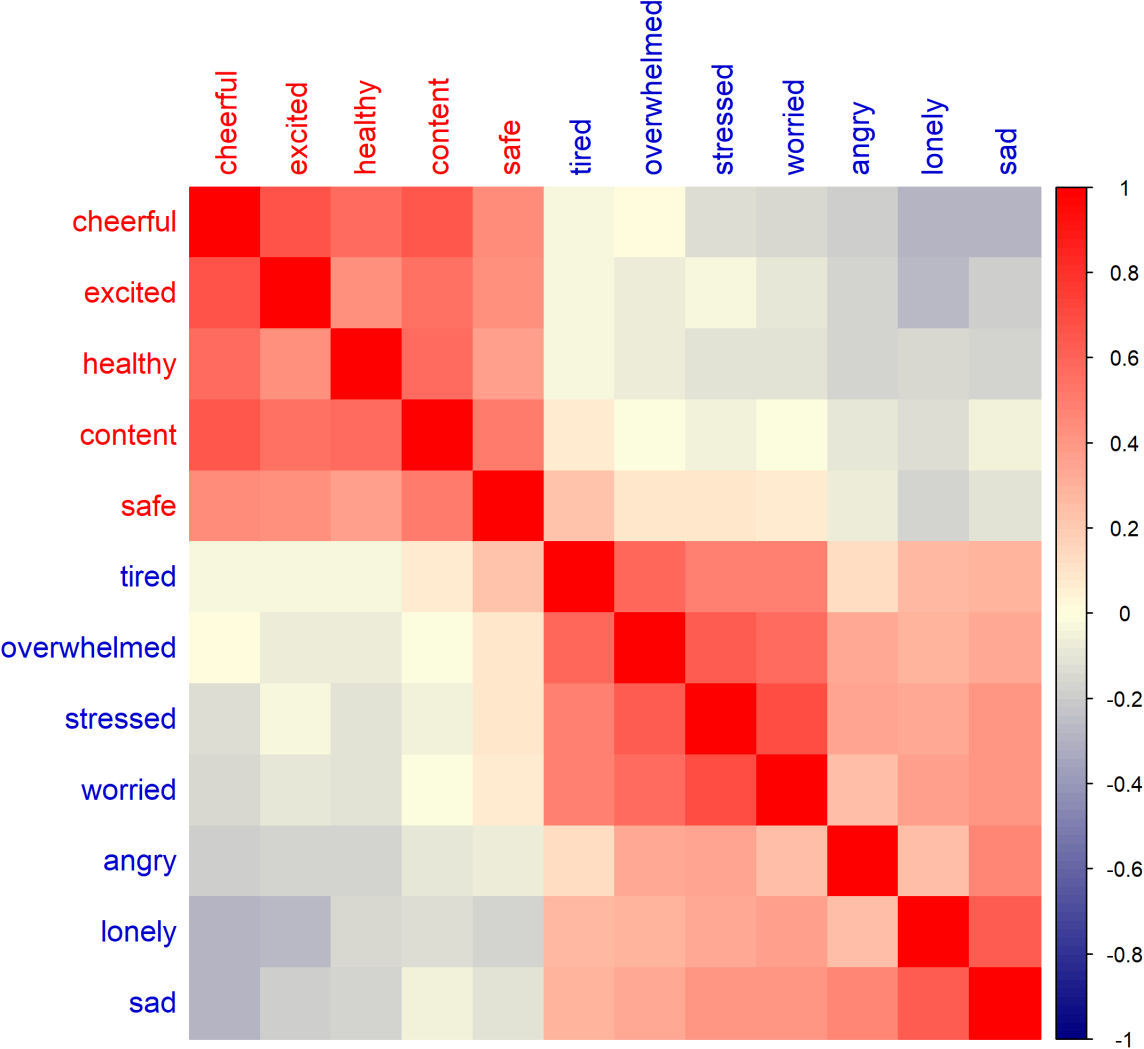
Heatmap of the pairwise correlations between the 12 emotions from the daily EMA data, where red indicates a strong positive correlation and blue indicates a strong negative correlation. Spearman correlation was used to measure the strength and direction of the monotonic relationship between the emotion variables.

Fig 1 presents a heatmap of the pairwise correlations between the 12 emotion measurements, illustrating the overall relationships among different emotional states. The figure highlights the contrasting patterns between positive and negative emotions, offering insights into their co-occurrence and differentiation. In the heatmap, positive emotions (e.g., cheerful, excited, safe) are expected to show strong positive correlations with each other, indicating that individuals who frequently experience one positive emotion are likely to experience others in the same category. Conversely, negative emotions (e.g., tired, overwhelmed, sad) also exhibit strong positive correlations among themselves but weakly negative correlations with positive emotions.

**Fig 2:**
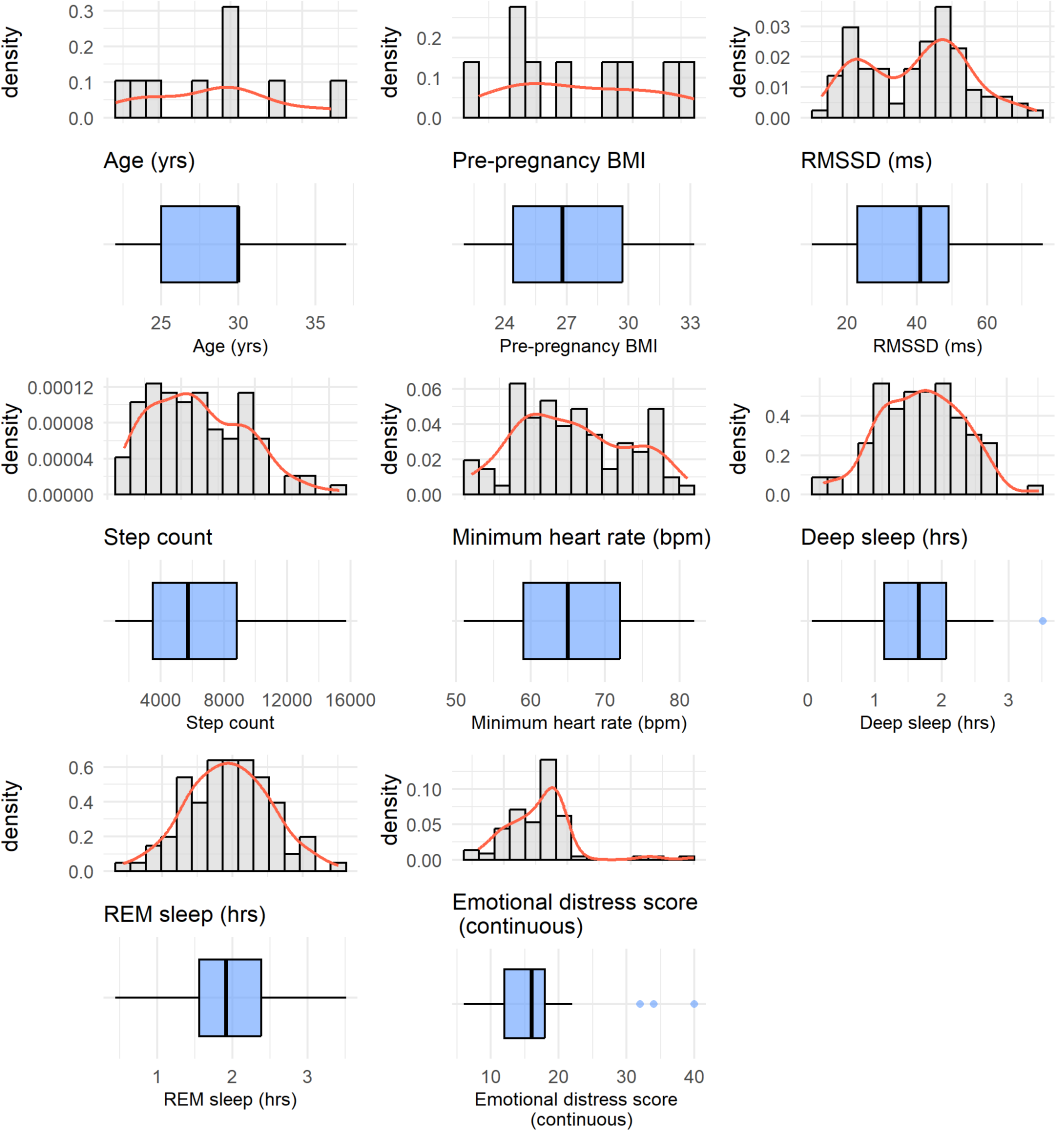
Distributions of variables considered in the model, presented as histograms and corresponding boxplots below. The histograms illustrate the estimated probability density of each variable, while the boxplots show the median, interquartile range, and potential outliers.

In order to quantify emotional distress, we constructed an overall emotional distress (ED) score based on the 12 emotions measured through EMA. Before summing the 12 emotions, we redefined the five positive emotions (cheerful, excited, healthy, content, and safe) into their negated forms (e.g., not cheerful, not excited), ensuring that all items consistently reflected some level of negative emotional experience. To achieve this, we reverse-coded the Likert scale for the five positive emotions so that 0 became 4, 1 became 3, 2 remained 2, 3 became 1, and 4 became 0. This transformation allowed for a consistent interpretation where higher scores across all 12 emotions indicated greater emotional distress. The adjusted emotion ratings were then summed, resulting in an overall ED score, with higher values reflecting greater emotional distress. As shown in Fig 2, this composite score ranged from 6 to 40.

To enhance interpretability, we categorized the overall ED score into categorical levels of emotional distress using quantile-based cutoffs, dividing the scores into 3 groups (i.e., using 34th and 67th percentiles). Based on this approach, we defined the cutoffs as follows: scores 6–14 as low emotional distress, 15–18 as moderate emotional distress, and 19–40 as high emotional distress. This resulted in 39%, 41%, and 20% of observations in the low, moderate, and high emotional distress level, respectively. This tertile approach balanced interpretability (“low/moderate/high” mapping) with sufficient representation across categories for stable estimates. Sensitivity analyses using alternative categorization schemes (binary splits and quartiles) yielded consistent results, with higher emotional distress showing a stronger positive association between physical activity and HRV.

### Statistical analysis

#### Primary outcome of interest.

In this study, we examined potential factors that influence physiological stress among pregnant women. We used the root mean square of successive differences (RMSSD) as the objective measure for heart rate variability (HRV), which serves as an indicator of physiological stress. The RMSSD is calculated by taking the square root of the mean of the squares of successive differences between adjacent heartbeats. This measure is particularly useful for assessing the parasympathetic nervous system’s activity, which is responsible for promoting relaxation and recovery [38]. Higher RMSSD values indicate greater variability in heart rate, reflecting a more adaptable and resilient autonomic nervous system, which is often associated with lower physiological stress levels [6]. Conversely, lower RMSSD values suggest reduced variability and higher physiological stress levels, and a greater risk for cardiovascular and other health-related issues [39,40]. In this paper, we use RMSSD and HRV interchangeably to refer to measures of autonomic regulation and cardiovascular health.

#### Modeling.

We examined the relationships between RMSSD, physiological and emotional factors (e.g., physical activity and emotional distress) using the generalized estimating equations (GEE) model, given the longitudinal nature of the data [41]. Due to repeated measurements from a limited number of subjects, subject-wise correlations on measured outcomes were expected to play a significant role in data analysis. Parameter estimations could be biased if within-subject correlations are ignored. The GEE approach, which accounts for the correlated structure of the data, produces more precise statistical analysis through a robust estimation of standard errors [41]. We compared several within-subject correlation structures (independent, exchangeable, and first-order autoregressive, AR(1)) using the quasi-likelihood under the independence model criterion (QIC) and chose the one with the lowest QIC value [42]. This process identified the AR(1) correlation structure as the best fit, which we used in the final GEE model.

Additionally, we used the log-transformed RMSSD as the response variable in the model. The analysis was performed using R software, and the GEE model was computed using the ‘geepack’ package. Moreover, we adjusted for five physiological and demographic variables in the model, including age, pre-pregnancy BMI, minimum resting heart rate, and deep and rapid eye movement (REM) sleep. These covariates were included based on their known associations with physiological stress, with prior research indicating their potential effects on HRV [24–27], and were adjusted for in the analysis to account for potential confounding.

## Results

Participants (**N* *= 9) were primarily Hispanic women between the ages of 22 and 37 years, with an average age of 28.8 years (SD = 4.65) and an average pre-pregnancy BMI of 27.4 (SD = 3.84). Summary statistics of the variables included in the GEE models are provided in Table 1 and their distributions are provided in Fig 2.

**Table 1:** Summary statistics for continuous variables considered in the model presented as mean ± standard deviation and range, and for categorical variables as count (%) for *N* = 9 subjects with a total of 93 measurements.

Variable	Mean ± SD or Count (%)	Range
Age (yrs)	28.8 ± 4.7	(22, 37)
Pre-pregnancy BMI	27.4 ± 3.8	(22, 33.2)
RMSSD (ms)	38.8 ± 15.9	(10, 76)
Daily step count	6,135 ± 3,211	(1,125, 15,718)
Heart rate minimum (bpm)	65.5 ± 8.0	(51, 82)
Deep sleep (hrs)	1.63 ± 0.66	(0.06, 3.52)
REM sleep (hrs)	1.94 ± 0.60	(0.44, 3.51)
Daily emotional distress level:		
Low	36 (39%)	–
Moderate	39 (41%)	–
High	18 (20%)	–

We first examined the individual effects of physical activity and emotional distress on HRV while adjusting for age, pre-pregnancy BMI, resting heart rate, and sleep parameters (deep and REM sleep). Since the response variable in our GEE models is the log-transformed RMSSD, all interpretations in this section are based on exponentiated coefficients. As shown in Table 2, holding all other variables constant, every additional 1,000 steps was associated with a 1.3% increase in RMSSD (e^0.000013*1000^ = 1.013; *P*-value = 0.039), suggesting that greater physical activity is associated with improved autonomic function (95% CI: 0.064%, 2.57%).

**Table 2:** Model results examining the effects of emotional distress level (low, moderate, or high) and physical activity (step count) on HRV (log-transformed RMSSD), adjusting for covariates. Low emotional distress level was the reference level.

	Estimate	Std. Err	Wald	*P*-value
*(Intercept)*	7.06	0.191	1369.29	< 0.001 ***
Age (yrs)	-0.0415	0.0044	88.11	< 0.001 ***
Pre-pregnancy BMI	-0.0076	0.0027	8.07	0.0045 **
**Step count**	0.000013	0.0000063	4.25	0.039 *
Heart rate minimum (bpm)	-0.0352	0.0023	244.96	< 0.001 ***
Deep sleep (hrs)	0.1000	0.0240	17.47	< 0.001 ***
REM sleep (hrs)	-0.0509	0.0129	15.65	< 0.001 ***
**Moderate emotional distress**	0.0034	0.0404	0.01	0.9331
**High emotional distress**	0.0116	0.0323	0.13	0.7180

Significance codes: 0 ‘***’ 0.001 ‘**’ 0.01 ‘*’ 0.05 ‘.’ 0.1 ‘’ 1.

Moreover, emotional distress levels showed distinct associations with HRV (see Table 2). As mentioned earlier, emotional distress was categorized into three levels: low, moderate, and high, with low emotional distress being the reference level in our model. Compared to low emotional distress, moderate emotional distress was associated with a 0.3% increase in RMSSD (95% CI: -7.3%, 8.6%), while high emotional distress was associated with a 1.2% increase in RMSSD (95% CI: -5.0%, 7.8%). However, neither of these associations was statistically significant, indicating that emotional distress alone may not have a strong main effect on HRV.

Therefore, we included an interaction term between step count (physical activity) and emotional distress levels to examine whether the effect of physical activity on HRV varies by emotional distress level. The results in Table 3 revealed a statistically significant interaction between step count and high emotional distress (*P*-value < 0.001), suggesting that the relationship between physical activity and HRV depends on emotional distress level. Specifically, for subjects experiencing high emotional distress, each additional 1,000 steps was associated with a 3.5% increase in RMSSD (95% CI: 2.6%, 4.4%) compared to those experiencing low emotional distress. Thus, although the overall (main) effect of physical activity on RMSSD was not significant, there was a significant interaction with distress level (*p*-value < 0.001), indicating that physical activity was positively associated with RMSSD only among individuals reporting high emotional distress (3.5%; 95% CI: 2.6%, 4.4%). Physical activity had little to no association with RMSSD for those with moderate distress (0.6%; 95% CI: -0.7%, 1.9%) and low distress (0.6%; 95% CI: -0.4%, 1.5%).

**Table 3:** Model results examining the moderating effect of emotional distress level (low, moderate, or high) on the relationship between physical activity (step count) and HRV (log-transformed RMSSD), adjusting for covariates. Low emotional distress level was the reference level.

	Estimate	Std. Err	Wald	*P*-value
*(Intercept)*	7.10	0.2390	881.28	< 0.001 ***
Age (yrs)	-0.0413	0.0051	66.30	< 0.001 ***
Pre-pregnancy BMI	-0.0089	0.0025	12.43	< 0.001 ***
Step count	0.0000055	0.0000049	1.27	0.2606
Heart rate minimum (bpm)	-0.0347	0.0025	188.28	< 0.001 ***
Deep sleep (hrs)	0.1050	0.0265	15.77	< 0.001 ***
REM sleep (hrs)	-0.0520	0.0149	12.21	< 0.001 ***
Moderate emotional distress	-0.0334	0.0461	0.53	0.4684
**High emotional distress**	-0.2050	0.0346	35.05	< 0.001 ***
Step count × Moderate emotional distress	0.0000063	0.0000067	0.90	0.3419
**Step count × High emotional distress**	0.000035	0.0000045	59.76	< 0.001 ***

Significance codes: 0 ‘***’ 0.001 ‘**’ 0.01 ‘*’ 0.05 ‘.’ 0.1 ‘’ 1.

**Fig 3:**
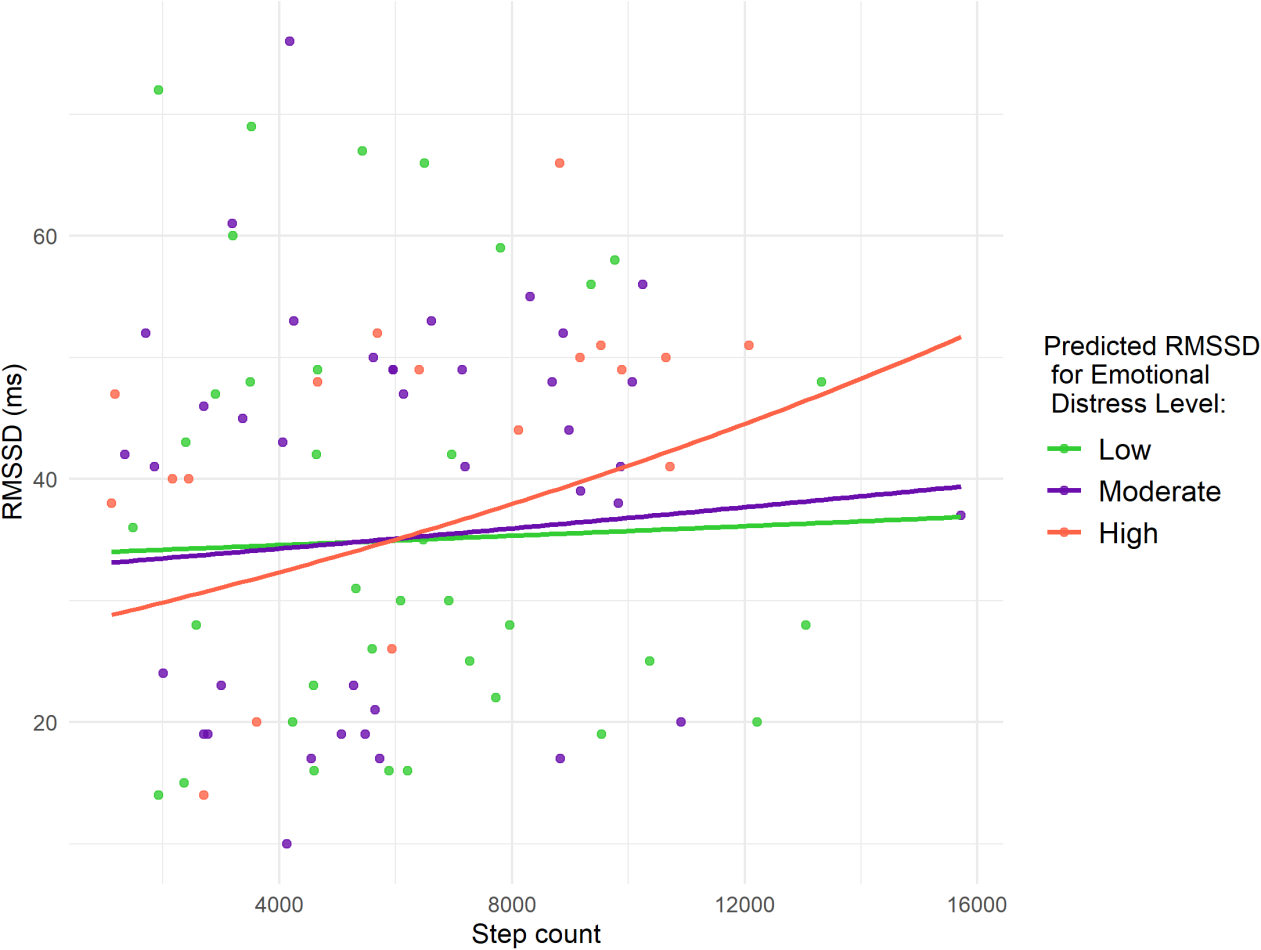
Predicted RMSSD as a function of daily step count at different levels of emotional distress for a pregnant woman with average age, pre-pregnancy BMI, minimum heart rate, and deep and REM sleep. Points represent observed daily measurements for the nine pregnant women, color-coded by emotional distress level.

Fig 3 visually illustrates the heterogeneous effects of daily step count on HRV across different emotional distress levels, according to the model presented in Table 3. The figure depicts the predicted RMSSD as a function of daily step count at different levels of emotional distress for a pregnant woman with average values for age, pre-pregnancy BMI, resting heart rate minimum, and sleep measures (deep and REM), as summarized in Table 1. This visualization highlights how the relationship between physical activity and HRV differs depending on emotional distress levels while holding other covariates constant.

Additionally, the analysis indicated that high emotional distress was significantly associated with lower HRV compared to low emotional distress. Specifically, on days with no physical activity (zero daily steps), high emotional distress corresponded to a 19% reduction in RMSSD compared to low emotional distress days (95% CI: -23.8%, -12.8%). In contrast, moderate emotional distress did not show a significant association with HRV relative to low emotional distress under these conditions.

Several covariates were also significantly associated with RMSSD, and similar effects were observed for both models (with and without the interaction term). Older age was significantly associated with lower RMSSD, with each additional year of age corresponding to a 4.0% decrease in RMSSD (95% CI: -5.0%, -3.1%). Pre-pregnancy BMI was negatively associated with RMSSD, with each unit increase in BMI corresponding to a 0.9% decrease in RMSSD (95% CI: -1.4%, -0.4%). This is consistent with previous studies showing that higher BMI is associated with reduced parasympathetic nervous system activity, which lowers heart rate variability. Excess body weight can lead to chronic inflammation and increased sympathetic nervous system activity, both of which contribute to decreased RMSSD [43]. Higher resting heart rate was strongly associated with lower HRV, with each 1 bpm increase in heart rate corresponding to a 3.4% decrease in RMSSD (95% CI: -3.9%, -2.9%).

Regarding sleep, REM sleep was negatively associated with RMSSD, with each additional hour of REM sleep corresponding to a 5.1% decrease in RMSSD (95% CI: -7.8%, -2.3%). This is likely because during REM sleep, the body’s “rest and digest” activity is lower and the “fight or flight” response is higher compared to deeper sleep stages, which results in lower heart rate variability [44]. In contrast, deep sleep was positively associated with RMSSD, where each additional hour of deep sleep corresponded to a 11.1% increase in RMSSD (95% CI: 5.5%, 17%). These observed effects were statistically significant across all models.

## Discussion

In our study, we hypothesized that emotional distress moderates the relationship between PA and HRV. In other words, instead of assuming that the association between PA and HRV is uniform regardless of the participant’s emotional state, we hypothesized that the positive association is stronger on high-distress days. This aligns with the buffering effect framework [16], which suggests behaviors like PA can help protect the body from the harmful effects of emotional distress.

One key mechanism involves vagal tone, which is a measure of how well the vagus nerve regulates heart rate and promotes calm, restful states [17]. Higher vagal tone reflects stronger parasympathetic (i.e., relaxation) activity and better ability to recover from stress. When emotional distress is high, vagal tone tends to be lower, indicating reduced capacity to manage stress [18]. PA stimulates the nervous system and can boost vagal tone, helping the body “bounce back” from stress [19]. On days with greater emotional distress, there is more room for improvement, so the increase in HRV after PA may be larger than on low-distress days. In other words, PA may more effectively counteract distress-induced reductions in HRV by strengthening vagal tone and restoring the balance between stress and recovery systems [17–19].

Our findings align with prior research demonstrating that emotional distress exacerbates reductions in HRV during pregnancy. For example, one study reported significant declines in HRV during the first and second trimesters, with more pronounced reductions in pregnant women experiencing higher distress levels [15]. This study used a linear mixed model with subjective emotions as predictors and HRV as the response, but did not include PA as a predictor. In addition, emotional distress was assessed weekly and dichotomized into broad frequency categories, which may introduce recall bias and reduce sensitivity to short-term fluctuations. By contrast, our study utilized EMA to capture daily, real-time emotional states in conjunction with objective wearable data, thereby minimizing recall bias and facilitating the analysis of daily dynamics. Consistent with prior findings, we observed that high emotional distress was associated with lower HRV, particularly on less active days, reinforcing the detrimental impact of distress on autonomic function. However, such an association was not observed for low or moderate emotional distress levels, demonstrating the importance of considering the interaction between physical activity and emotional state when interpreting distress-related reductions in HRV.

Furthermore, although existing research generally supports PA’s beneficial effects on HRV and emotional distress, our findings emphasize that the stronger positive association between PA and HRV was primarily observed under conditions of high emotional distress. This suggests that physical activity may help preserve autonomic function when psychological demands are high, rather than offering uniform benefits across all levels of distress. A prior study examining prenatal yoga reported increases in HRV and reductions in emotional distress, suggesting a regulatory role of PA in autonomic function [10]. While consistent with our results, that study did not test emotional distress as a moderator of the PA-HRV relationship, and therefore could not address whether PA is particularly beneficial under heightened distress. Moreover, their focus on structured activity (yoga) contrasts with our use of naturalistic daily PA (step counts), which captures real-life variability in intensity and context.

Similarly, another study found that regular PA was associated with lower resting heart rate and higher HRV during pregnancy, further supporting PA’s role in cardiovascular adaptation [9]. However, that study emphasized physiological outcomes without examining emotional distress as a potential mechanism or moderator. In addition, a separate study suggested that PA may be protective against emotional distress during pregnancy [45], but the focus was primarily on psychological outcomes rather than linking PA, distress, and HRV in an integrated framework. By bridging these domains, our study contributes a novel perspective by showing that PA can mitigate the adverse impact of distress on HRV, particularly during periods of heightened distress. This integrative approach suggests that PA not only supports general cardiovascular adaptation but also buffers against stress-related autonomic dysfunction in everyday contexts.

### Implications

Our findings have important implications for intervention strategies aimed at improving maternal health. The association between PA and improved HRV was strongest on days when emotional distress was elevated. This suggests that PA may be especially helpful for managing physiological stress on more emotionally difficult days. This points to the potential value of tailoring activity-based strategies to individuals experiencing higher levels of distress. The growing availability of mHealth technologies, such as wearable devices, enables real-time monitoring of HRV and PA patterns, which could inform personalized recommendations responsive to both emotional and physiological states. However, we acknowledge that the broader use of these tools and their impact on health outcomes need to be tested in larger and more diverse groups. Given the increasing integration of mHealth tools in prenatal care, future research should also explore how digital interventions can optimize PA engagement to improve maternal cardiovascular health, especially for individuals experiencing high levels of emotional distress.

### Limitations

This study has some limitations to consider when interpreting the results. First, the sample size was small, with only 9 pregnant women recruited through convenience sampling. Although the longitudinal design yielded a total of 93 observations (10 observations per participant, on average), which allowed us to capture day-to-day variation, the small sample size limits the stability of estimated interaction effects and reduces statistical power. As a result, effect size estimates may be inflated, confidence intervals might be wider, and individual participants with more longitudinal observations might disproportionately influence the findings. Therefore, the results should be interpreted as exploratory and hypothesis-generating rather than confirmatory.

Second, the demographic composition of our sample (majority Hispanic) strengthens research equity by including an underrepresented population, but it also limits generalizability. Cultural and contextual factors, such as norms around physical activity, cultural views of emotional distress, stress-coping strategies, daily environments, and familiarity with mHealth technologies, may shape both behavior and physiological responses [46–49]. Because EMA surveys in this study captured only emotional ratings and did not include broader contextual information, we were unable to examine these influences in detail. These factors could affect how physical activity, emotional distress, and HRV are related in other cultural, racial, socioeconomic, or demographic contexts, so these findings are considered context-specific and need to be confirmed in larger and more diverse populations.

Lastly, the use of objective measures from the Oura ring and subjective measures from EMA data may introduce potential biases. Participants have access to their Oura ring data through their smartphone app, which could potentially influence their behavior and compromise data accuracy. The subjective nature of the emotional data collected through EMA also carries the potential for bias, as emotions may be influenced by factors beyond the study’s scope. Although EMA is subjective and may introduce bias, we assessed a broad range of emotions and aggregated them into a composite emotional distress score, which provides a more stable emotional profile. We also recognize that increasing the frequency of EMA prompts throughout the day in future studies could further minimize subjective bias and improve data quality.

### Future directions

Some concrete directions for future research are as follows. First, we recommend adding cortisol measurements, such as saliva samples for daily stress levels or hair samples for longer-term stress exposure, to examine how physical activity and emotional distress interact across different stress biomarkers. Combining cortisol and HRV data would provide a more comprehensive picture of how psychological and physiological stress are regulated daily during pregnancy. Second, future studies should include larger and more diverse groups, especially participants from underrepresented racial and socioeconomic backgrounds, to test whether the relationship we observed varies across different populations. Finally, we suggest designing experimental studies that actively prompt physical activity during high-distress moments, such as through mobile alerts or reminders, to test whether physical activity in those moments helps reduce stress responses in real-time. These steps would help clarify when and for whom physical activity is most helpful in managing daily stress.

## Conclusion

While prior studies have examined physical activity and emotional distress in relation to HRV separately, most have relied on static, cross-sectional measures that overlook how daily changes in emotional distress may affect the physiological effects of physical activity on HRV. In pregnancy, a period marked by continuous shifts in stress and behavior, our findings demonstrate that integrating longitudinal physiological and emotional data via mHealth tools can reveal within-person dynamics not captured by static assessments. Specifically, we found that physical activity was associated with higher HRV on days when participants reported greater emotional distress, highlighting a moderating effect of emotional distress. This association was minimal on days with low or moderate emotional distress, suggesting that physical activity may be particularly beneficial on days with high emotional distress. These results indicate that increasing daily physical activity could help reduce physiological stress among emotionally distressed pregnant women, based on observed associations, and may inform the design of targeted interventions. However, given our small and relatively homogeneous sample, these findings should be considered preliminary. They nonetheless provide promising evidence of a nuanced relationship between physical activity, emotional distress, and the autonomic nervous system, warranting replication in larger and more diverse cohorts.
